# Computer Skills and Electronic Health Records (EHRs) in a State Tertiary Hospital in Southwest Nigeria

**DOI:** 10.3390/epidemiologia4020015

**Published:** 2023-04-27

**Authors:** Maureen Nokuthula Sibiya, Oluwatoyin Rhoda Akinyemi, Olanrewaju Oladimeji

**Affiliations:** 1 Division of Research, Innovation and Engagement, Mangosuthu University of Technology, Durban 4031, South Africa; sibiya.nokuthula@mut.ac.za; 2 Department of Health Sciences, Faculty of Health Science, Durban University of Technology, Durban 4001, South Africa; 3 Department of Epidemiology and Biostatistics, School of Public Health, Sefako Makgatho Health Sciences University, Pretoria 0208, South Africa

**Keywords:** computer skills, adoption, EHR, ICT, healthcare workers, hospital, patient management

## Abstract

Adopting change is something that is often resisted, as is often the case in the adoption of electronic health records (EHRs) in healthcare organizations. Embracing this will require computer knowledge to handle the system for the management of patients and their care. This study aims to determine the computer skills required for the uptake of electronic health records (EHR) by healthcare workers in an annex of the state teaching hospital, Okela Health Centre (OHC) Ado-Ekiti. The study uses a cross-sectional research design with a structured questionnaire distributed to 30 healthcare workers across seven disciplines working in the hospital. Descriptive statistics of frequency tables and percentages were used to ascertain the relationship between computer skill usage and the adoption of EHR. The majority of respondents were only efficient in Microsoft Word (MW), email, and WhatsApp, with efficiency rates of 63.4%, 76.6%, and 73.3%, respectively. The majority were not efficient in Microsoft Excel (ME) and Microsoft Access (MA), at 56.7% and 70%, respectively. Computer appreciation is an important basis for the adoption of EHR in hospitals.

## 1. Introduction

The healthcare data stored as electronic health records need to be updated from time to time. Patient information, such as previous history, must be readily available anytime and anywhere to support treatment. Such stored information requires highly skilled manpower with information communication and technology (ICT) which must be continuously maintained. The healthcare organization is required to manage the right of access to the data alongside its retrieval and any communication [1].

Several data are generated from the activities of healthcare workers in the process of patient management. These must be stored and processed in an efficient way to allow for quick access and dependable storage of the information [2]. The work of healthcare providers, decision makers, and policy makers is largely dependent on their use of quality data in health information systems to ensure an effective, efficient, and reliable healthcare service. Several poor data qualities, due to integrating data from various sources, have been reported as reasons for the non-adoption of electronic health records (EHRs). This invariably leads to different data structures which makes extracting useful information from the data difficult. Quality data help the earnings of the hospital. They also help to ensure smooth processing and planning in healthcare services. Therefore, having a reliable and dependable quality of data cannot be overemphasized to guide against false conclusions and wrong decisions. EHRs will assist a great deal in ensuring structured data entry and smart interfaces [3]. The EHR system is regarded as a cornerstone that facilitates the integration of several e-health techniques among the various health information technology (HIT) initiatives in the international healthcare system. Many countries tried to create an EHR system, resulting in a 46% increase over the last five years. Nevertheless, over half of the world’s electronic record-keeping programs fell short of their objectives. Particularly, the adoption of EHRs continues to be a significant obstacle for health services in low-income nations. Only 15% of low-income nations have nationalized the use of electronic record-keeping in medical facilities [4]. Due to the digital divide and other socioeconomic concerns, including electrical power failures, healthcare providers who are resistant to using new technology, and ICT infrastructure, sub-Saharan countries appear to be behind in implementing these technologies [4]. Computer literacy could be described as the computer-associated knowledge required to acquire, communicate, process, and comprehend the fundamental knowledge needed to make sound health decisions [4]. Electronic health records (EHRs) have been gaining popularity around the word but are still experiencing slow adoption in underdeveloped and developing countries. EHRs refer to patient information that is stored digitally to eliminate the practice of manual registries and repositories. It provides secure storage, secure information exchange, and is made available to different levels of healthcare practitioners [5]. It could also refer to a digital repository of patient data that is kept safe and is readily available for numerous authorized personnel. Adopting EHRs in hospitals offers many benefits, such as minimized costs, increased revenues, improved patient care, reduced filing space, enhanced confidentiality of data, a reduction in medical and dispensing errors, centralized patient data management, and allows for sharing medical information among members of a health team [6,7].

Despite the great benefits, many hospitals are yet to embrace EHRs in the management of their patients. In research of the primary factors that affect healthcare professionals’ willingness to adopt an EHR system, computer literacy was discovered to be one of them [4]. Healthcare in developed countries has adopted various technologies which are used to carry out several activities. Information is transmitted and transferred between healthcare providers in this manner. Communication relies largely on various information technologies that are still evolving. The electronic medication management assistant (eMMA) is an example of this. It is a laboratory prototype for a mobile app aimed at assisting patients with their prescribed medication on a constant schedule to adhere to drug therapy. This employs a conversational user interface (CUI) to notify users to take their medications. It is mainly intended to help the patient at home prior to their admission as an inpatient as well as following their discharge from rehabilitation [8]. Research was carried out in South Africa on the theoretical framework for the adoption of patient record management systems with the goal of determining the variables that influence the exchange of patient information among physicians, which stimulates the advancement of diagnosis. In this case, slow adoption of EHRs was seen to be contributing to diagnosis errors. Data contained in electronic systems are desired and may be utilized by a variety of medical specialists, including hospitals, laboratories, pharmacies, government agencies, employers, healthcare institutions, academic research institutions, and public health organizations. Various groups have advocated for an increase in the use of ICT in healthcare organizations [9,10]. Around the world today, there are several healthcare information systems in existence, and these are described in Table 1 below.

### 1.1. Factors Affecting the Adoption of EHRs

The factors that affect the adoption of EHRs include attitudes toward EHRs, perceived usefulness, perceived ease of use, social influence, computer self-efficacy, potential threat to physician autonomy, and confidentiality concerns.

- Attitude Toward EHRs

This refers to a patient’s positive or negative feelings about engaging in the desired behavior. One’s mindset toward using technology is a critical indicator in the technology acceptance/adoption model. In implementing an EHR system, effort must be concentrated on developing peoples’ attitude towards its use [19].

- Perceived Usefulness

This is the extent to which people believe that adopting an EHR system will increase their job performance [20].

- Perceived Ease of Use

This considers whether individuals believe that using the system will require less effort. According to TAM, user satisfaction is a significant component in both attitudes toward technology use and its perceived benefits [21].

- Social Influence

The influence of peers and colleagues regarding the use of a system significantly affects the number of people who may then go on to use it. If a person believes that individuals who are significant to him or her are using the system, his or her involvement is likely to be swayed toward its adoption. Coworkers and peers, as well as top management, are examples of people who can have a social influence on healthcare teams’ acceptance of EHR systems [22].

- Computer Self-Efficacy

The ability to use a computer quite well determines his/her efficacy. Healthcare providers who have high computer self-efficacy are more likely to use EHRs than their counterparts who do not. As technology advances, it becomes necessary for healthcare providers to be computer compliant, as this has become a requirement for employment in several organizations. Following that, a constant on-the-job training program can be planned to refresh people’s computer knowledge prior to EHR implementation to boost its acknowledgement [22].

- Potential Threat to Physician Autonomy

Physicians are distinguished by their high level of professional independence. This is when an individual assumes that by employing a specific system, he or she would lose control over the circumstances, methods, procedures, or content of his or her work, which is untrue. Though opposition is likely due to the significant changes occurring in EHR implementation, which may influence leadership roles or power dynamics in medical practice, this should not influence their choice to utilize EHRs [23].

- Confidentiality Concerns

It is a source of wider issue, especially in developing countries, that EHRs could be accessed by unauthorized persons. Some individuals are concerned about the confidentiality of patient information, which is one of the reasons they are hesitant to adopt EHRs [24]. Having relevant rules and guidelines in place, as well as informed consent from patients, could serve as safeguards against concerns about confidentiality.

### 1.2. Possible Effects of Poor Management of EHRs on Patient Care Management

During research on the implementation and feasibility of an electronic health record-integrated patient-reported outcomes symptom and needs monitoring pilot in ambulatory oncology, an EHR system was discovered to be a helpful technique for patient maintenance and appointment reminders. Patients received a reminder via their patient portal to complete an assessment on Epic MyChart. Epic MyChart is a tool used to complete assessment through a previously stated preferred mode (i.e., via e-mail, MyChart patient portal message, or phone call). Evaluations were accessible in English and Spanish and took approximately 8–10 min to complete through the patient’s MyChart account (web or smartphone app). Patients were instructed to finalize these evaluations 72 h prior to their next appointment. Without adequate knowledge of healthcare staff on computer use, this may result in missing appointments which could have been corrected through messages, emails, and calls to the patients at least 72 h before the appointment. There is also a possibility of work overload as staff will be forced to do this on behalf of patients at their walk-in time [25].

Another effect of poor implementation of EHRs on patient care management is waste of resources. Some institutions committed to implementing an EHR system financially no matter how small its use may be, hence the need for effective use to prevent a waste of resources. Sustainability of the system is imperative and may be ensured through the training of healthcare professionals, a change of attitude, and the right mindset on the part of end-users [26].

Automated clinical data generated for policy making, disease incidence definition, and care outcomes will be disorganized, thereby forfeiting the impact of EHRs on patient care management [27].

The issue of training cannot be overemphasized. Khan S.Z et al. (2012) opines that EHRs will not be effective if there is no necessary training. The technical complexity involved in the use of EHRs demands a high level of technical competency on the part of the healthcare provider in order to prevent information use from becoming a burden when the EHR system is put in place. This also applies when considering poor preparation and use of data [28].

Having considered the various factors affecting the adoption of EHRs, there is a need to highlight the aim of the study, which is to determine the need for computer skills as a determinant in the adoption of EHRs in Okela Health Centre (OHC), an annex of Ekiti State University Teaching Hospital Ado-Ekiti, in southwest Nigeria.

## 2. Materials and Methods

**Figure 1:**
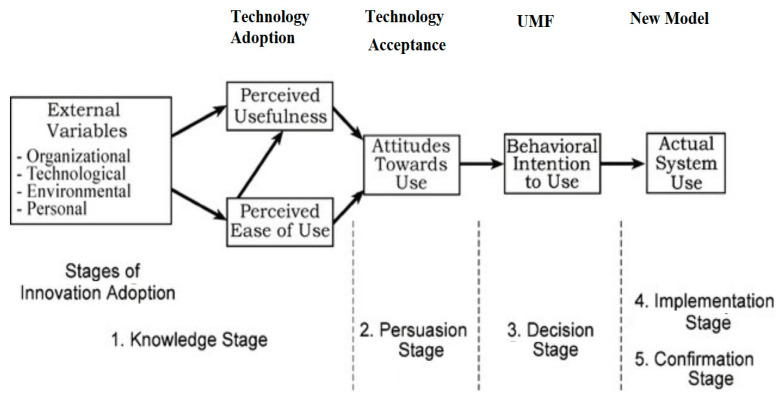
Adopting EHRs through the use of the TAM model. Source: author designed.

Adopting EHRs, as with other technologies, may be determined and explained by the use of the technology acceptance model (TAM) which is depicted in Figure 1 below:

Using the TAM diagram, when a technology is introduced, the first stage involves assessment of the organizational system already in use. The technological aspects are then considered, which are the probable tools and equipment used. The next step includes assessing the environmental buildings and the personal aspects, including an evaluation of their knowledge on the use of EHRs, which in this case is computer skills. This is followed by the knowledge stage. The unfreeze-move-freeze system gradually moves away from their paper-based method previously being used, encouraging people towards adopting the new system (move) and final implementation of the adopted system before it is frozen back [29].

**Figure 2:**
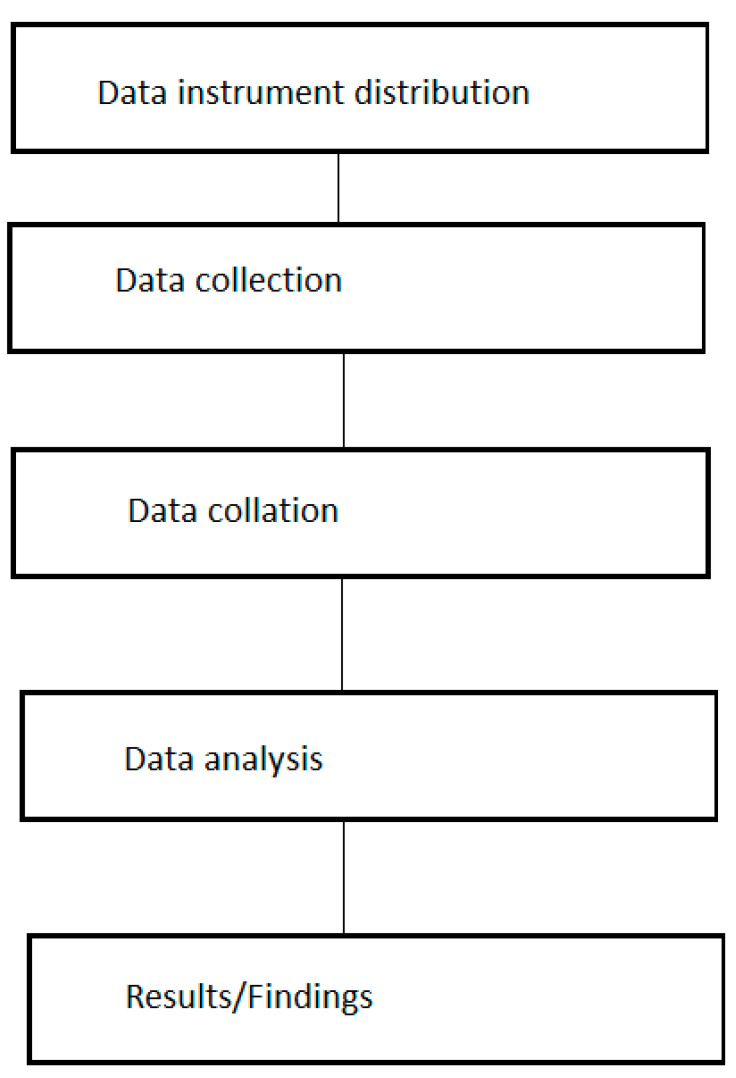
Diagram of the methodology employed.

The methodology employed in the study as shown in Figure 2 are: data instrument distribution (in this case which is the questionnaire), data collection, data collation, analysis of the data and lastly extracting the results or the findings of the study.

A structured questionnaire was self-designed and self-distributed to the entire population of healthcare professionals working at OHC Ado Ekiti, which comprises of 4 nurses, 13 community health extension workers, 5 attendants, 1 health assistant, 1 pharmacy technician, 1 laboratory technician, 3 doctors, and 2 health information management technicians, totaling to 30 respondents. The questionnaires were designed to include questions regarding their demographic information and respondents were also asked to rate their level of efficiency from ‘not at all efficient’ to ‘extremely efficient’ in the usage of some computer functions, such as MS Excel, MS Word, MS Access, email, and WhatsApp. These categories of healthcare workers were given the questionnaires to fill, which was self-administered by the researcher to the respondents at their duty post and was retrieved after completion. The study, which was conducted in early 2023, lasted approximately 2 weeks in order to be able to capture staff off-duty during the first week that the study began. The paper-based questionnaires were taken to the study area to be self-administered by the researcher. The questionnaires were retrieved and returned after being filled. The retrieved questionnaire was subjected to analysis using frequency tables and percentages.

## 3. Results

The results from the analyzed questionnaire are shown below, where ME means Microsoft Excel, MW means Microsoft Word, and MA means Microsoft Access.

**Figure 3:**
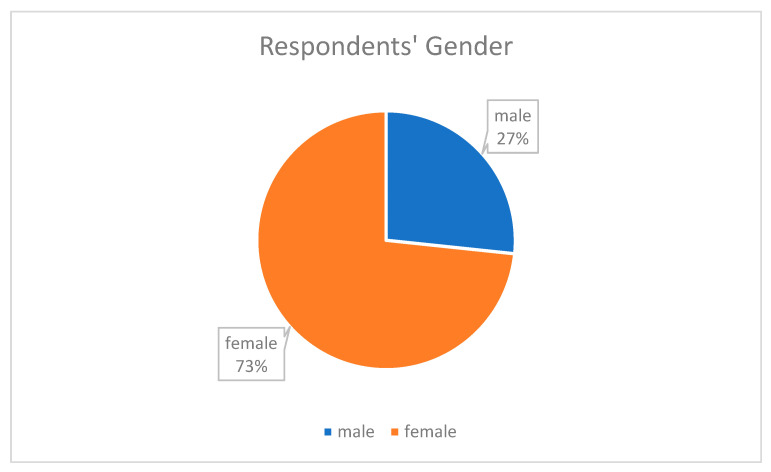
Pie chart of respondents’ gender.

**Figure 4:**
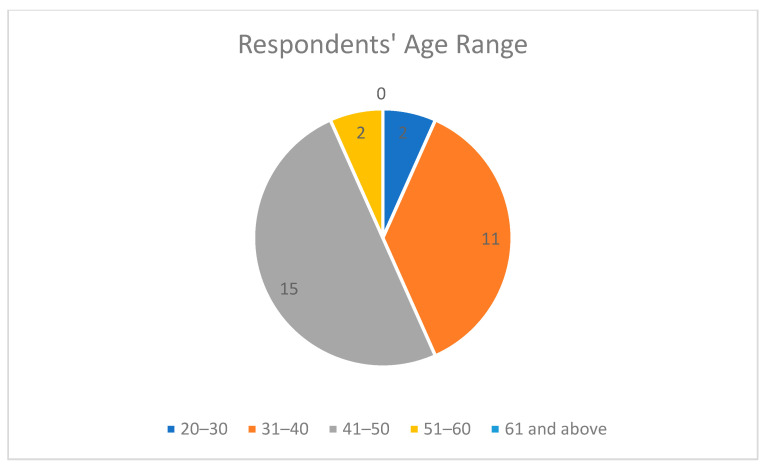
Pie chart for respondents’ age range.

**Figure 5:**
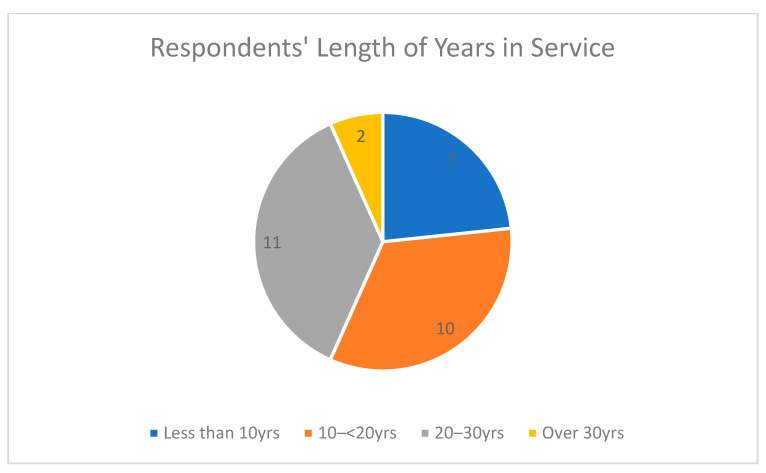
Pie chart for respondents’ length of years in service.

The demographic information of the respondents showed that the highest percentage of the respondents were females at 73.3% as shown in Figure 3, and people in the age range 41–50 provided the most responses at 50% as shown in Figure 4, which means they are young and vibrant people who can easily grab new knowledge when introduced to it. A total of 36.7% were between 20–30 years in their length of service, which showed that they had a good number of years of experience as shown in Figure 5. The community health extension workers (CHEW) had the highest number of respondents with regards to job title as shown in Table 2.

Respondents’ level of skills in ME is depicted in Table 3, and it is shown that majority of the respondents, approximately 56.7%, were not efficient in the use of ME, while 43.3% were efficient. Mostly EHR platforms are formed of spreadsheets, hence the need for staff to develop this computer skill to enhance smooth adoption of EHRs into their work for the management of their patients. Table 4 shows the respondents’ level of skills in MW. The majority of the respondents (63.4%) were efficient in the use of MW, while 36.6% were not efficient. A total of 70% of the respondents were not efficient in the use of MA while the remaining 30% were efficient, as shown in Table 5. Table 6 shows the respondents’ level of skills in email usage, and the highest percentage of 76.6% confirms their efficiency in its use while 23.4% were not efficient. Table 7 depicted the respondents’ level of skills in WhatsApp, where 73.3% of the total respondents were efficient and 26.7% were not efficient. From the above findings, it is evident from the percentage of people that were not efficient in the use of these few computer functions that implementing an EHR system for management of patients will be difficult. This highlights the need for prioritizing computer literacy as part of the requisite for employment and also the need to train and retrain those already in the system for its use.

## 4. Discussion

This result is consistent with the findings on the study conducted on artificial intelligence healthcare service resource adoption by medical institutions based on the technology organization environment (TOE) framework, where computer processing power, computer experience, computer knowledge, perceived usefulness of intelligent systems, and their ease of use are seen as factors influencing the adoption of intelligent healthcare services by medical institutions with integrated medical care [30].

All healthcare professionals, including nurses, health information management practitioners, doctors, laboratory scientists, pharmacists, and all other members in healthcare teams, are expected to have extensive knowledge and be competent in the use of computer systems for the management of their patients’ healthcare using EHRs. Adopting EHRs will largely depend on the computer skill usage of the end users before it can optimally be put into use. In the light of the huge data requirements and improved patient care, there is a pressing need for all healthcare professionals to acquaint themselves with adequate computer appreciation in order to meet the with the needs of improved healthcare services through electronic health appliances, which are fast growing around the world [31,32,33]. Since the largest number of respondents of the study were within their active age and had long years of working experience, they should be encouraged to undertake independent computer appreciation training and more on-the-job training.

## 5. Conclusions

There is a need for continuous training and retraining of existing staff, and computer literacy must be emphasized as part of the requisite for future employment. This will ensure the smooth running of EHRs in hospitals. Computer appreciation and its knowledge are great determinants for the adoption of EHRs, and if it is going to be introduced into practice there is need for all staff, no matter their job title, to become acquainted with it.

Limitation of the study: This study is limited to an annex of the main hospital EKSUTH, because they are yet to utilize the EHR system unlike the main hospital itself. Future studies may be undertaken on the applicability of computer appreciation on practical use and the challenges encountered with this. Research may also focus on the other annex of the hospital.

## Figures and Tables

**Table 1:** Examples of healthcare information systems in existence worldwide.

S/N	Country	Electronic Care	Care Type
1.	South Africa	South African neighborhood HIV treatment hospital employing a digitalized pharmacy monitoring system [11].	HIV
2.	Kenya	Ailment observation and sports medicine using information technology [12].	Injury Observation
3.	Uganda	Knowledge of electronic healthcare record implementation [13].	HIV
4.	Ghana	In rural areas, incorporating essential occasion enrolment, verbal post-mortem, and electronic medical records [14].	Essential Enrolment
5.	Mozambique	HIV treatment and monitoring of patients receiving ART [15].	MozART
6.	Korea	Regional networking system used to merge data from various health institutions for the purpose of having a database that is secured and dependable for use in medical services and treatment. Ensures patient information is controlled and shared for easy access to optimal care wherever and whenever [16].	Networking information system
7.	Germany	Used for the prediction of individualized prognosis and treatment response in order to make a well-informed decision [17,18].	Cloud-based healthcare IoT

**Table 2:** Respondents’ job titles.

Job Title	Number	Percentage
HIMO	0	0
HIMT	2	6.7
Nurses	4	13.3
Doctors	3	10
Pharmacist	1	3.3
Laboratory technician	1	3.3
Radiographer	0	0
CHEW	13	43.3
Health attendants	5	16.7
Health assistant	1	3.3
Total	30	100

**Table 3:** Respondents’ level of skills in MS Excel.

	Frequency	Percentage (%)
Not at all efficient	15	50
Moderately not efficient	2	6.7
Efficient	6	20
Moderately efficient	4	13.3
Extremely efficient	3	10
Total	30	100

**Table 4:** Respondents’ level of skills in MS Word.

	Frequency	Percentage (%)
Not at all efficient	7	23.3
Moderately not efficient	4	13.3
Efficient	8	26.7
Moderately efficient	3	10
Extremely efficient	8	26.7
Total	30	100

**Table 5:** Respondents’ level of skills in MS Access.

	Frequency	Percentage (%)
Not at all efficient	15	50
Moderately not efficient	6	20
Efficient	2	6.7
Moderately efficient	3	10
Extremely efficient	4	13.3
Total	30	100

**Table 6:** Respondents’ level of skills in email.

	Frequency	Percentage (%)
Not at all efficient	5	16.7
Moderately not efficient	2	6.7
Efficient	6	20
Moderately efficient	3	10
Extremely efficient	14	46.6
Total	30	100

**Table 7:** Respondents’ level of skills in WhatsApp.

	Frequency	Percentage (%)
Not at all efficient	2	6.7
Efficient	6	20
Moderately efficient	5	16.7
Extremely efficient	17	56.6
Total	30	100

## Data Availability

Not applicable.
